# Frail older people with decreased cognition can perceive reduced self-determination in self-care and social relationships

**DOI:** 10.1186/s12877-023-04492-y

**Published:** 2024-01-03

**Authors:** Isabelle Andersson Hammar, Theresa Westgård, Synneve Dahlin-Ivanoff, Katarina Wilhelmson

**Affiliations:** 1 Department of Health and Rehabilitation, Institute of Neuroscience and Physiology, The Sahlgrenska Academy, University of Gothenburg, Box 455, 405 30 Gothenburg, Sweden; 2 The Gothenburg University Centre for Ageing and Health (AgeCap), Gothenburg, Sweden; 3 Department of Psychiatry and Neurochemistry, Institute of Neuroscience and Physiology, The Sahlgrenska Academy, University of Gothenburg, Gothenburg, Sweden; 4 Department of Occupational Therapy and Physiotherapy, Sahlgrenska University Hospital, Gothenburg, Region Västra Götaland Sweden; 5 Department of Acute Medicine and Geriatrics, Sahlgrenska University Hospital, Gothenburg, Region Västra Götaland Sweden

**Keywords:** Cognition, Cross-sectional study, Decision-making, Frail elderly

## Abstract

**Background:**

Self-determination in old age is essential for people’s experiences of good subjective health and quality of life. The knowledge concerning how frail older people with decreased cognition perceive their ability to be self-determined in the different dimension in daily life is, however, limited. The aim of this study was therefore to explore the relationship of self-determination and cognition in frail older people.

**Methods:**

This study was a cross-sectional secondary data analysis using baseline data with 119 frail people 75 ≥ from a larger randomized control trial. Self-determination was measured with the statements from the *Impact on Participation and Autonomy-Older persons* (IPA-O). Cognition was measured using the Mini Mental State Examination (MMSE), where decreased cognition was broadly defined as a score below 25 points. Fisher’s exact test was used to test differences in proportions of perceiving self-determination in relation to cognition. The Relative Risk (RR) with a 95% Confidence Interval (CI) was used to explore the risk of perceiving reduced self-determination in relation to cognitive functioning.

**Results:**

Nearly the entire study population, regardless of cognitive functioning, perceived self-determination in *Financial situation*. For people with decreased cognition, the relative risk for perceiving reduced self-determination was statistically significant higher in activities related to *Self-care* and in *Social relationships* when comparing with the participants with intact cognition.

**Conclusion:**

Perceiving self-determination when being old, frail and having decreased cognition is possible but is dependent upon which activities that are involved. Organizing healthcare needs according to the older people’s wants and wishes is crucial regardless of people having a cognitive decline or not when the effort is to enable the people to be as self-determined as they want. The frail older people with decreased cognition should be treated as being experts in their own lives, and healthcare professionals should navigate the older people to get to their desired direction.

**Trial registration:**

ClinicalTrials.gov, NCT02773914. Retrospectively registered 16 May 2016.

## Introduction

Being able to decide in one’s own life, i.e. exercising self-determination, is both natural and fundamental regardless of age. Exercising self-determination has been linked to positive health [1, 2], and an improved quality of life [3]. Self-determination can be defined as a process of having control and ethical/legal rights, and ability and knowledge to make decisions of a person’s free choice [4]. Internationally, health and social care have shifted during the last decades towards strengthening people’s self-determination concerning their own health and social care by several acts and movements [5–9]. Two recent movements designed to help older people to identify their own health priorities, and then to align their care with their priorities highlights the importance of involving older people in their care process and in making decisions. These evidenced-based frameworks are the Age-Friendly Heath System [8, 9] and the Patient Priorities Care (PPC) approach [10, 11]. The Age-Friendly Health System [8, 9] is a framework built up on the four M’s *what matters*, *medication*, *mentation* and *mobility*. *What matters* focuses on what is important for the older people regarding their own healthcare and their daily life, with the goal to create a patient-oriented care plan. *Medication,* focuses on using only age friendly medication when needed, which means using medication that does not affect what matters to the older people, their mentation, and their mobility. *Mentation*, focuses on preventive care and management directly towards the older people’s mental health, with regards to delirium, dementia and depression. Finally, *mobility*, focuses on older peoples possibilities to continue moving safely, which in turn enables for them to do what matters in their lives [9]. The PPC approach [10, 11] moves from focusing on only the management of individual diseases, towards helping older people identify their own health priorities. This approach [10, 11] recognizes that patients are the experts on their own health, and know best what they want from their healthcare. On the other hand, healthcare professionals are the experts on how to get the patients in the right direction. Delivering care based on the PPC approach [10, 11] will result in a reduction of unwanted and unhelpful care and medications while supporting appropriate health and community-based care, as suggested by the Age Friendly Health System [8, 9].

Focusing on the older people’s priorities and what matters to them is important when delivering evidence-based, high-quality of care [9, 10]. However enabling self-determination in people is easier in theory than in real life practice. This is especially crucial for people with high age, who need help when performing daily activities [12]. When frailty and reduced cognition are added to the puzzle, older people’s possibilities to exercise self-determination may be endanger to an even greater extent. Frailty is a geriatric syndrome that is related to a deterioration of multiple physiological systems in high age [13], that often results in decreased abilities to perform daily activities and morbidity [14]. Knowledge concerning to what extent frail older people with decreased cognition exercising self-determination in diverse dimensions in their lives is sparse. Having this knowledge could be important when developing care plans for older people with decreased cognition, so they can perceive that their care is self-determined friendly. Previous research [15] has shown that cognitively intact, community-dwelling older people living in Sweden experienced their self-determination as a shifting process, from governing oneself, to being governed by others. Reduced possibilities to govern were identified when the relationship and the communication between the older people and professionals were lacking [15]. Moreover, an earlier study [16] identified that older people living in nursing homes in Finland who were cognitively intact experienced barriers due to their physical frailty, declines in daily activities, and because of health care professionals’ and older people’s attitudes towards them. When the older people controlled when they went to bed, got dressed, and controlled activities related to privacy and social life with their relatives, their own free will was realized [16]. Further, another study [17] showed that people with decreased cognitive functioning living in Australia had a desire to remain an important part of decision making regarding their own life for as long as possible, despite what the future might be. The older people wanted support that was subtle, and to be assisted while they still were able to make decisions. When health and home carers forced decision upon them, the older people felt marginalised and excluded [17]. Self-determination in old age is, thus, a universal phenomenon that has been studied in several countries. However, there is still a gap in comprehending how frail older people with decreased cognition experience their self-determination in different dimension in daily life. Therefore, the aim of this study was to explore the relationship of self-determination and cognition in frail older people.

## Methods

### Design

This study was a cross-sectional secondary data analysis using baseline data from the larger CGA-Swed study [18], which was a randomized control trial with frail older people (75 +) living in the west coast of Sweden. The study was approved by the Regional Ethical Review Board in Gothenburg, ref. no: 4899–15. Trial Registration: ClinicalTrials.gov, NCT0277391. The study also followed the ethical principles for medical research involving human subjects – The WMA Declaration of Helsinki [19].

### Sample and procedure

In this study, the sample consisted of baseline data with a total of 119 people 75 years or older, in need of an unplanned hospital admission to a medicine or geriatric ward, that were screened as frail in accordance with the FRESH-screening instrument [18, 20]. Potential participants were invited to join the study by the care coordinator in the emergency department. Both verbal and written information regarding the study was provided. Potential participants were informed of their right to withdraw from the study at any time, that all data would be handled confidentially, and that no individuals could be identified. Those who agreed to participate signed a consent form. Some of the participants had decreased cognitive functioning making them unable to understand the information well enough to give their consent. In these cases, their next of kin signed the consent form. The care coordinator was usually the one who determined the cognitive impairment. People admitted via fast track (stroke, coronary infarct, or hip fracture) were excluded from the CGA-Swed study [18]. For detailed information regarding the CGA-Swed study, see the study protocol [18].

### Data collection

Data were collected by using a structured questionnaire with the older people during their hospital stay. In a few cases, the participants were discharged prior to their baseline interview. Therefore, a few of the baseline interviews were performed in the participants homes shortly after discharge. The researchers, registered occupational therapists, physiotherapists, nurses, and physicians, performed all the interviews and measurements at baseline. All were trained in observing and assessing in accordance with the guidelines for the specific outcome measurements.

Participant’s perceived *self-determination* was assessed with the statements from the Swedish Impact on Participation and Autonomy- Older persons (IPA-O) [21], that consist of a total of 22 items divided in seven dimensions and a summery item as follows: *mobility* (4 items), *self-care* (5 items), *activities in and around the house* (4 items), *financial situation* (1 item), *use of time* (1 item), *social relationship* (5 items), *help and support others* (1 item), and a *summary item* concerning *My chances to live the way I want are good* (1 item). The statements can be answered with totally agree, partly agree, neither agree nor disagree, disagree and totally disagree [21]. The IPA-O [21] has previously been tested for validity and test–retest reliability for people 70 years and older, showing good psychometrical properties. *Cognition* was measured with the Mini Mental State Examination (MMSE) [22]. For detailed information regarding measures of baseline characteristics, see the study protocol [18].

### Statistics

Descriptive statistics was used to explore baseline characteristics. Prior to analysing the data, the measures of self-determination and cognition were dichotomized. Regarding self-determination, the response option *totally agree* was dichotomized to *perceiving self-determination,* and the remaining four response options (partly agree, neither agree nor disagree, disagree, and totally disagree) were dichotomised to *perceiving reduced self-determination*. Regarding cognition, a cut-off below 25 points was used as an indicator of decreased cognition, in accordance with previous publications from the CGA-Swed study [18, 23, 24]. Fisher’s exact test was used to test differences in proportions of perceived self-determination between people with intact cognition and people with decreased cognition. A *p*-value of 0.05 or less was considered significant. The Relative Risk (RR) with a 95% Confidence Interval (CI) was used to explore the risk of perceiving reduced self-determination among people with intact cognition and people with decreased cognition. All calculations were performed per protocol. Statistical analyses were performed using IBM SPSS Statistics for Windows, version 26.0 (Armonk, NY: IBM Corp, 2019).

## Results

### Characteristics of participants

The sample consisted of 119 frail older people with the mean age of 86 years (range 75–100 years). Of this, 59 percent were female, 65 percent were living alone, and 34 percent rated their health as good. The majority of the sample were dependent in I-ADL, and 36 percent were dependent in P-ADL. Of the 116 participants who had been cognitive screened, 45 percent had decreased cognition (Table 1). Baseline characteristics of participants are presented in Table 1.

**Table 1:** Baseline characteristics of participants (*n*=119)

	*n *(%)
Age	
Mean	86
Range	75-100
Female	70 (59)
Living alone	77 (65)
Tertiary education^a^	18 (15)
Dependence in I-ADL^b^	107 (90)
Dependence in P-ADL^c^	43 (36)
Good self-rated health^d^	41 (34)
Decreased cognition^e^	52 (45)^f^

^a^Tertiary education (initiated and completed university or college)

^b^Dependent in at least one instrumental activity of daily living

^c^Dependent in at least one personal activity of daily living

^d^Excellent/very good/good

^e^Cognition <25 points measured with the Mini Mental State Examination (MMSE)

^f^*n*=116

Dependence in I-ADL was pronounced in participants with both intact and decreased cognition. Nearly the entire sample, regardless of cognition, was screened as having morbidity/disability (a score of 3 or more in any category, on the rating scale) (Table 2).

**Table 2:** Characteristics of participants with intact and decreased cognition divided in degree of dependence and morbidity/disability (*n*=116)

	Intact cognition (*n*=64) *n *(%)	Decreased cognition (*n*=52) *n *(%)
Dependence in I-ADL^a^	56 (88)	48 (92)
Dependence in P-ADL^b^	18 (28)	24 (46)
Morbidity/disability^c^	60 (94)	51 (98)

^a^Dependent in at least one instrumental activity of daily living

^b^Dependent in at least one personal activity of daily living

^c^Being screened as having morbidity/disability (a score of 3 or more in any category, on the rating scale)

#### Perceptions of self-determination in people with intact and decreased cognition

A few of the participants were unable to answer all the items in the IPA-O, resulting in a few internal dropouts (Tables 2, 3, 4, 5 and 6). The majority of the participants perceived a high degree of self-determination concerning their *Financial situation*; 94 percent among participants with intact cognition respectively 92 percent among those with decreased cognition. A high degree of self-determination was also shown in *Use of time*, with 84 percent among participants with intact cognition respectively 83 percent among participants with decreased cognition. Approximately a third of the sample, i.e. both people with intact and decreased cognition, perceived lower levels of self-determination in *Mobility* (28 percent respectively 27 percent), and in *Social relationships* (33 percent in both groups) (Table 3).

**Table 3:** Number and percentage distribution of perceiving self-determination^a^ on dimension level among frail older people with intact and decreased cognition (*n*=116)

	Intact cognition (*n*=64) *n *(%)	Decreased cognition (*n*=52) *n *(%)
Mobility (4 items)	18 (28)	14 (27)
Self-care (5 items)	51 (80)	30 (58)
Activities in and around the house (4 items)	29 (45)	22 (42)
Financial situation(1 item)	60 (94)	48 (92)
Use of time (1 item)	54 (84)	43 (83)
Social relationship (5 items)	21 (33)	17 (33)
Help and support others (1 item)	28 (44)	19 (37)
Summary (1 item)	24 (38)	21 (40)

^a^Perceiving self-determination (totally agree)

#### The relationship between perceiving reduced self-determination and cognitive functioning

The results showed statistically significant reduced perceptions of self-determination (*p* = 0.044, RR = 2.38) within the dimension *Self-care*—*My chances to decide when I get washed and dressed are good* (item 6), among participants with decreased cognition in contrast to participants with intact cognition (Table 4). In the dimension *Social relationship—My chances to talk to people close to me on equal terms are good* item (item 16), the participants with decreased cognition also had statistically significant reduced perceptions of self-determination (*p* = 0.041, RR = 3.71) (Table 5).

**Table 4:** The proportion (%), *p*-value and the Relative Risk (RR) with a 95 % Confidence Interval (CI) of perceiving reduced self-determination in frail older people with intact and decreased cognition (*n* = 116)

	Intact cognition (*n*=64)		Decreased cognition (*n*=52)		
	*n *(%)	RR	*n *(%)	RR (95 % CI)	*p-*value
Mobility (4 items)					
My chances to decide…					
1. where to get around in my house are good	1 (2)	1	3 (2)	3.69 (0.40-34.46)	0.252
2. when I want to get around in my house are good	3 (5)	1	5 (10)	2.09 (0.52-8.34)	0.296
3. when to visit relatives and friends are good	22 (34)	1	22 (42)	1.28 (0.81-2.03)	0.294
4. to go on the sort of trips/holidays I want to are good	45 (70)	1	36 (69)	0.99 (0.78-1.25)	0.922
Self-care(5 items)					
My chances to decide…					
5. to get washed and dressed the way I want are good	10 (16)	1	11 (22)^4^	1.38 (0.64-2.99)	0.414
6. when I get washed and dressed are good	7 (11)	1	13 (26)^3^	2.38 (1.03-5.51)	**0.044**
7. when I want to go to bed or get up are good	7 (11)	1	8 (16)^3^	1.46 (0.57-3.76)	0.430
8. when I want to go to the toilet and when I need to are good	1 (2)	1	4 (8)^3^	5.12 (0.59-44.39)	0.138
9. when I want to eat and drink are good	7 (11)	1	10 (20)^2^	1.87 (0.77-4.55)	0.170
Activities in and around the house (4 items)					
My chances to get…					
10. light tasks done around the house, either by myself or by others the way I want are good	6 (9)	1	9 (18)^4^	1.88 (0.72-4.49)	0.199
11. heavier tasks done around the house, either by myself or by others the way I want are good	27 (42)	1	17 (34)^3^	0.81 (0.50-1.30)	0.379
12. housework done, either by myself or by others when I want are good	22 (34)	1	21 (62)^3^	1.22 (0.76-1.95)	0.403
13. minor repairs and maintenance work done in my house, and garden either by myself or by others the way I want are good	15 (25)^5^	1	14 (30)^1^	1.21 (0.65-2.25)	0.545

Significant values are marked with bold type

^1^*n*=47

^2^*n*=49

^3^*n*=50

^4^*n*=51

^5^*n*=61

**Table 5:** The proportion (%), *p*-value and the Relative Risk (RR) with a 95 % Confidence Interval (CI) of perceiving reduced self-determination in frail older people with intact and decreased cognition (*n* = 116)

	Intact cognition (*n*=64)		Decreased cognition (*n*=52)		
	*n *(%)	RR	*n *(%)	RR (95 % CI)	*p-*value
Financial situation (1 item)					
14. My chances to choose how I spend my own money are good	4 (6)	1	3 (6)^3^	0.94 (0.22-4.02)	0.935
Use of time (1 item)					
15. My chances to use leisure time the way I want are good	10 (16)	1	8 (16)^3^	1.00 (0.43-2.36)	0.993
Social Relationship (5 item)					
16. My chances to talk to people close to me on equal terms are good	3 (5)^5^	1	9 (18)^3^	3.71 (1.06-12.98)	**0.041**
17. The respect I receive from people who are close to me are good	5 (8)^5^	1	8 (16)^2^	2.02 (0.70-5.78)	0.192
18. My chances to talk to acquaintances on equal terms are good	19 (30)^5^	1	24 (48)^2^	1.59 (0.99-2.56)	0.055
19. The respect I receive from acquaintances are good	15 (24)^4^	1	14 (29)^1^	1.21 (0.65-2.25)	0.557
20. My chances to see people as often as I want are good	32 (51)^5^	1	18 (36)^2^	0.71 (0.46-1.10)	0.127
Help and support others (1 item)					
21. My chances to help or support people in any way are good	34 (55)^4^	1	31 (62)^2^	1.13 (0.83-1.55)	0.442
Summary (1 item)					
22. My chances to live the way I want are good	39 (62)^5^	1	30 (59)^3^	0.95 (0.70-1.28)	0.739

Significant values are marked with bold type

^1^*n*=48

^2^*n*=50

^3^*n*=51

^4^*n*=62

^5^*n*=63

#### Perceptions of problems caused by health or disability in relation to cognitive functioning

No statistically significant differences with regards to perceptions of problems caused by health or disability in relation to cognitive functioning were found. In the dimension of *Mobility*, 82 percent of the participants with decreased cognition respectively 88 percent of the participants with intact cognition perceived problems caused by health or disability affecting their chances to be self-determined. Further, approximately 80 percent of the sample, regardless of level of cognition, perceived problems caused by health or disability affecting their chances to be self-determined in *Activities in and around the house* (Table 6).

**Table 6:** The proportion (%), *p*-value and the Relative Risk (RR) with a 95 % Confidence Interval (CI) of perceiving problems caused by health or disability within the seven IPA-O dimensions (*n *= 115)

	Intact cognition (*n*=64)		Decreased cognition (*n*=51)		
	*n *(%)	RR	*n *(%)	RR (95 % CI)	*p-*value
Mobility					
If your health or your disability affect your chances of getting around where and when you want, to what extent does this cause you problems?	56 (88)	1	42 (82)	0.94 (0.80-1.10)	0.450
Self-care					
If your health or your disability affect your self-care, to what extent does this cause you problems?	33 (52)	1	31 (61)	1.18 (0.85-1.63)	0.320
Activities in and around the house					
If your health or your disability affect your activities in and around the house, to what extent does this cause you problems?	50 (78)	1	41 (80)	1.03 (0.85-1.24)	0.765
Financial situation					
If your health or your disability affect the opportunities you have over spending your own money, to what extent does this cause you problems?	22 (34)	1	17 (33)	0.97 (0.58-1.62)	0.907
Use of time					
If your health or your disability affect how you use your time, to what extent does this cause you problems?	41 (64)	1	26 (51)	0.80 (0.57-1.10)	0.169
Social Relationship					
If your health or your disability affect your social relationships, to what extent does this cause you problems?	44 (70)^a^	1	33 (65)	0.93 (0.71-1.20)	0.564
Help and support others					
If your health problems or disability affect your opportunities to help and support others, to what extent does this cause you problems?	40 (63)^a^	1	32 (63)	0.99 (0.75-1.31)	0.935

^a^*n* = 63

## Discussion

The present study explored the relationship of self-determination and cognition in frail older people. The results showed that people with decreased cognition had more than two times higher risk of perceiving reduced self-determination in the *Self-care—people’s chances to decide when getting washed and dressed the way one wants*, compared to people with intact cognition. This result suggests that older people with decreased cognition perceived that activities related to deciding when to get washed and when to get dressed was not done as they wanted and wished. From a societal perspective, this vulnerable population could be supported by having an advance care planning, with the purpose to enable them to express their wishes for the future while they still possess mental capacity. Organizing care needs according to older people’s wants and wishes regardless of having a cognitive decline or not is crucial. People should be treated as being experts in what they want from their healthcare, and healthcare professionals should be the experts in how the older people might get there [10, 11]. Care-planning meetings that take place in people’s homes enable older people’s participation and involvement in the discussions, and they were able to influence their concerns relating to the amount of care, service and the choice of provider, however they were not able to influence the way the help should be provided or organised [25]. Regardless of a person’s cognition, a care plan should outline a person’s assessed care needs, and should meet the needs of these people. The care plans should be designed and prepared to give care services the recipient understands and agrees with it. In contrast, caregivers, family members, and healthcare providers may simply take over tasks which not only robs the old people of their independence, it also affects their self-worth [26]. This is supported by a recent study [27] that identified the need to evaluate the knowledge and attitude of the caregivers of people with dementia. Providing care to this population may be burdensome and strenuous because of the complexity of multiple interacting diseases and treatments. Moreover, it is also humanly hard, because care and illness can affect peoples’ views on life, how they live, and relate to others [28]. The Patient Priorities Care aims to ensure that care plans serve rather than hinder the lives of older people living with comorbidities [10, 29]. When this is achieved, perceptions of self-determination in frail older people with decreased cognition could be possible and achievable.

In the present study, people with decreased cognition had nearly four times higher risk of perceiving reduced self-determination in *Social relationships* when comparing with people with intact cognition*.* The frail older people in the present study with decreased cognition perceived that their *chances to talk to people close to them on equal terms* were not achieved. Self-determination is a process of having control and rights, and it also includes having the ability and knowledge to make decisions which are determined by a person’s free choice [4]. In the present study, the findings are suggestive that older people’s ability to communicate and converse so they perceive that they are listened to, and seen as an equal was reduced when having decreased cognition. This could be due to the fact that language and cognition are closely related to each other. Previous research [30] has shown that people with decreased cognition may have language performance deficits. The language deficits can appear prior to the cognitive decline [30]*. *Reduced perceptions of self-determination in some social contexts and interactions could, thus, be an accumulative outcome of these deficits.

Regardless of having intact or decreased cognition, the majority of the participants in the present study perceived they were self-determined in their *Financial situation* and *Use of time*. These skills could be reasoned as not requiring language, rather require working memory and attention, which are abstract and logical. In contrast, approximately a third of the participants perceived self-determination regarding activities related to *Mobility* and *Social relationships*. A previous study [3], with people aged 65–100 years performed in the northern part of Sweden, showed that approximately 70 percent of the sample experienced self-determination in *Use of time*, and half of the sample experienced self-determination in *Mobility*. Only a third of the sample experienced self-determination in *Social relationships* [3]. The divergence between the findings in the present study and earlier findings may be due to the characteristics of the sample in the present study, consisting of frail older people, the majority of the sample were dependent in I-ADL, and roughly more than half of the sample was also dependent in P-ADL. Lastly, half of the sample were screened as having decreased cognition.

People aged 75 years or older were included in the present study. An important reason for choosing the age of 75 years or older was that the authors wanted to include a sample of only frail people. Including younger people would probably result in a more heterogenous sample in terms of frailty with a mix of non-frail, pre-frail and frail older people. Further, in the hospital were the CGA-Swed study [18] was performed, the hospital has as a routine to screen people aged 75 years and older with the FRESH-screening instrument [18, 20]. Only a total of 119 frail older people participated in the present study. This is a rather small sample which is a limitation, but one must keep in mind that the participant were not only old, but also frail. They were also dependent on help when performing activities of daily living (I-ADL and P-ADL), and approximately half of the sample were assessed as having decreased cognitive functioning. Achieving a larger sample with a people that is as vulnerable as the sample in the present study would not be easy.

In the present study, the sample consisted of people with intact and decreased cognition. There are some ethical issues that may arise when including people with decreased cognition, and therefore, people with decreased cognition are often underrepresented in research. One ethical issue may be that people with decreased cognition have difficulties in understanding and giving informed consent. When this was the case in the present study, next of kin signed the consent form. Moreover, partaking in several measures and answering questions may be tiring. In these cases, the researchers were observant of signs that may indicate participants being exhausted. The MMSE [22] was used when assessing cognition. This assessment was originally designed to evaluate language among other cognitive abilities [22]. By nature, the MMSE [22] assessment is verbally oriented, and when using this assessment it is important to consider if incorrect answers on the assessment could have been caused by a person’s language impairments rather than their cognitive deficits [31]. While it was not the intention of this study to explore this phenomenon, it is known that cognitive and language abilities are suggestive of a connection between aphasia; which is a disorder of language and involves a deterioration of communication skills, and the level of a person’s cognitive impairment [32]. Since both of these abilities need comprehension and communication, it is difficult to tell whether a person’s deficits are due to decreased language, or cognitive abilities, or both [33].

In this cross-sectional secondary data analysis, several data analyses were performed. However, only a few statistically significant differences between participants with declined cognition in contrast to participants with intact cognition were found. The findings could be due to chance, which must be kept in mind when interpreting the findings in the present study. Finally, this study has a quantitative design using statements from the Swedish Impact on Participation and Autonomy- Older persons (IPA-O) [21], that consist of a total of 22 items divided in seven dimensions and a summery item. No in-depth interviews focusing on the older peoples perceived self-determination within the diverse dimensions were performed. More research is thus needed focusing on frail older people with declined cognitive functioning and their possibilities and barriers to perceive self-determination in the dimensions in daily life.

## Conclusions

Frail older people with decreased cognition perceived reduced self-determination in activities related to *Self-care* and *Social relationships.* Perceptions of self-determination was high both among people with intact cognition and among people with decreased cognition in the dimensions of *Financial situation* and *Use of time.* Hence, perceiving self-determination when being old, frail and having decreased cognition is possible but is dependent upon which activities that are involved. Self-determination in frail older people with decreased cognition might be viewed as dynamic, and may vary from being a utopia to being possible depending on if language and communication are part of the daily activities. Organizing healthcare needs according to the older people’s wants and wishes is crucial regardless of people having a cognitive decline or not when the effort is to enable the people to be as self-determined as they want. The frail older people with decreased cognition should be treated as being experts in their own lives, and healthcare professionals should navigate the older people to get to their desired direction.

## Data Availability

The dataset used and analysed in the current study is available from the corresponding author on reasonable request. The data concerning the participants that underlie the results reported in this study will be available to the scientific community, immediately after publication and up to 5 years, upon request from researchers who provide a methodologically sound proposal and attempt to achieve aims in the approved proposal. Data will be stored for 10 years at the University of Gothenburg to enable for review. Proposals should be directed to the corresponding author, isabelle.a-h@neuro.gu.se. To gain access, data requesters will need to sign a data access agreement. Data is covered by the Public Access to Information and Secrecy Act and a confidentiality assessment will be performed at each individual request. Permission from the University of Gothenburg, the Institute of Neuroscience and Physiology, has to be obtained before data can be accessed.

## Abbreviations

ADL: Activities of Daily Living
CIRS-G: Cumulative Illness Rating Scale-Geriatrics
CGA: Comprehensive Geriatric Assessment
IADL: Instrumental Activities of Daily Living
IPA: Impact on Participation and Autonomy-Older persons
MMSE: Mini Mental State Examination
PADL: Personal Activities of Daily Living
RCT: Randomized control trial; RR: Relative Risk
